# ﻿Two new species of *Diaporthe* (Diaporthaceae, Diaporthales) associated with *Camelliaoleifera* leaf spot disease in Hainan Province, China

**DOI:** 10.3897/mycokeys.102.113412

**Published:** 2024-02-27

**Authors:** Hong Y. Liu, Dun Luo, Han L. Huang, Qin Yang

**Affiliations:** 1 Forestry Biotechnology Hunan Key Laboratory, Central South University of Forestry and Technology, Changsha 410004, China; 2 The Key Laboratory for Non-Wood Forest Cultivation and Conservation of the Ministry of Education, Central South University of Forestry and Technology, Changsha 410004, China; 3 Guangxi State-owned Bobai Forest Farm, Yulin 537600, China

**Keywords:** DNA phylogeny, systematics, taxonomy, tea-oil tree, two new taxa

## Abstract

Tea-oil tree (*Camelliaoleifera* Abel.) is an important edible oil woody plant with a planting area over 3,800,000 hectares in southern China. Species of *Diaporthe* inhabit a wide range of plant hosts as plant pathogens, endophytes and saprobes. Here, we conducted an extensive field survey in Hainan Province to identify and characterise *Diaporthe* species associated with tea-oil leaf spots. As a result, eight isolates of *Diaporthe* were obtained from symptomatic *C.oleifera* leaves. These isolates were studied, based on morphological and phylogenetic analyses of partial ITS, *cal*, *his3*, *tef1* and *tub2* gene regions. Two new *Diaporthe* species (*D.hainanensis* and *D.pseudofoliicola*) were proposed and described herein.

## ﻿Introduction

Tea-oil tree, *Camelliaoleifera* Abel., is a unique woody edible oil species in China, mainly distributed in the Qinling-Huaihe River area. It has a long history of cultivation and utilisation for more than 2300 years since ancient China (Zhuang 2008). Camellia oil, obtained from *C.oleifera* seeds, is rich in unsaturated fatty acids and unique flavours and has become a rising high-quality edible vegetable oil in China. The edible of tea-oil is also conducive to preventing cardiovascular sclerosis, anti-tumour, lowering blood lipid, protecting liver and enhancing human immunity (Wang et al. 2007). According to the Three-year Action Plan for Accelerating the Development of oil tea Industry, Hainan Province is listed as a key development area of oil tea and the total area of oil tea planting in the Province is planned to reach 16,667 hm^2^ by 2025. The development of *C.oleifera* industry is of great significance for the economic development of Hainan Province and the poverty alleviation of local farmers.

The expanding cultivation of *C.oleifera* over the last several decades has attracted increasing attention from plant pathologists to infectious diseases on this crop. Therein, diseases caused by *Diaporthe* species have become the emerging Camellia leaf diseases in southern China (Zhou and Hou 2019; Yang et al. 2021). During July and August of 2022, new leaf spots were detected on tea-oil tree with irregular, brownish-grey lesions, often associated with leaf margins. Infected leaves cultured on medium had dark pycnidia producing ellipsoid guttulate conidia, similar to that of *Diaporthe* species (Yang et al. 2021). The asexual morph is characterised by ostiolate conidiomata, with cylindrical phialides producing three types (alpha, beta and gamma conidia) of hyaline, aseptate conidia (Udayanga et al. 2011; Gomes et al. 2013).

Species identification criteria in *Diaporthe* has mainly relied on host association, morphology and culture characteristics (Mostert et al. 2001; Santos and Phillips 2009; Udayanga et al. 2011), which resulted in the description of over 200 species. Some species of *Diaporthe* were reported to colonise a single host plant, while other species were found to be associated with different host plants (Santos and Phillips 2009; Diogo et al. 2010; Santos et al. 2011; Gomes et al. 2013). In addition, considerable variability of the phenotypic characters was found to be present within a species (Rehner and Uecker 1994; Mostert et al. 2001; Udayanga et al. 2011). During the past decade, a polyphasic approach, based on multi-locus DNA data, morphological, phytopathological and phylogenetical analyses, has been employed for species boundaries in the genus *Diaporthe* (Huang et al. 2015; Gao et al. 2016, 2017; Guarnaccia and Crous 2017, 2018; Guarnaccia et al. 2018; Yang et al. 2018, 2020, 2021; Cao et al. 2022; Bai et al. 2023; Zhu et al. 2023).

The classification of *Diaporthe* has been on-going; however, little is known about species able to infect *C.oleifera*. Thus, the objective of the present study was to identify the prevalence of *Diaporthe* spp. associated with tea-oil tree leaf spot in the major plantations in Hainan Province, based on morphological and phylogenetic features.

## ﻿Materials and methods

### ﻿Fungal isolation

Leaves of *C.oleifera* with typical symptoms of leaf spots were collected from the main tea-oil camellia production fields in Hainan Province. Small sections (3 × 3 mm) were cut from the margins of infected tissues and surface-sterilised in 75% ethanol for 30 s, then sterilised in 5% sodium hypochlorite for 1 min, followed by three rinses with sterilised water and finally dried on sterilised filter paper (Yang et al. 2021). The sections were then plated on to PDA plates and incubated at 25 °C. Fungal growth was examined daily for up to 7 d. Isolates were then transferred aseptically to fresh PDA and purified by single-spore culturing (Fan et al. 2015). All fungal isolates were placed on PDA slants and stored at 4 °C. Specimens and axenic cultures are maintained in the Central South University of Forestry and Technology (CSUFT) in Changsha, Hunan Province.

### ﻿Morphological and cultural characterisation

Agar plugs (6 mm diam.) were taken from the edge of actively growing cultures on PDA and transferred on to the centre of 9 cm diam. Petri dishes containing 2% tap water agar supplemented with sterile pine needles (PNA; Smith et al. (1996)) and potato dextrose agar (PDA) and incubated at 25 °C under a 12 h near-ultraviolet light/12 h dark cycle to induce sporulation as described in recent studies (Gomes et al. 2013; Lombard et al. 2014). Colony characters and pigment production on PNA and PDA were noted after 10 d. Colony colours were rated according to Rayner (1970). Cultures were examined periodically for the development of ascomata and conidiomata. The morphological characteristics were examined by mounting fungal structures in clear lactic acid and 30 measurements at 1000× magnification were determined for each isolate using a Leica compound microscope (DM 2500) with interference contrast (DIC) optics. Descriptions, nomenclature and illustrations of taxonomic novelties are deposited in MycoBank (Crous et al. 2004a).

### ﻿DNA extraction, PCR amplification and sequencing

Genomic DNA was extracted from colonies grown on cellophane-covered PDA using a CTAB (cetyltrimethylammonium bromide) method (Doyle and Doyle 1990). DNA was estimated by electrophoresis in 1% agarose gel and the quality was measured using the NanoDrop 2000 (Thermo Scientific, Waltham, MA, USA), following the user manual (Desjardins et al. 2009). PCR amplifications were performed in a DNA Engine Peltier Thermal Cycler (PTC-200; Bio-Rad Laboratories, Hercules, CA, USA). The primer set ITS1/ITS4 (White et al. 1990) was used to amplify the ITS region. The primer pair CAL228F/CAL737R (Carbone and Kohn 1999) was used to amplify the calmodulin gene (*cal*) and the primers CYLH4F (Crous et al. 2004b) and H3-1b (Glass and Donaldson 1995) were used to amplify part of the histone H3 (*his3*) gene. The primer pair EF1-728F/EF1-986R (Carbone and Kohn 1999) was used to amplify a partial fragment of the translation elongation factor 1-α gene (tef1). The primer set T1 (O’Donnell and Cigelnik 1997) and Bt2b (Glass and Donaldson 1995) was used to amplify the beta-tubulin gene (*tub2*); the additional combination of Bt2a/Bt2b (Glass and Donaldson 1995) was used in case of amplification failure of the T1/Bt2b primer pair. The PCR amplifications of the genomic DNA with the phylogenetic markers were undertaken using the same primer pairs and conditions as in Yang et al. (2018). PCR amplification products were assayed via electrophoresis in 2% agarose gels. DNA sequencing was performed using an ABI PRISM 3730XL DNA Analyzer with a BigDye Terminater Kit v.3.1 (Invitrogen, USA) at the Shanghai Invitrogen Biological Technology Company Limited (Beijing, China).

### ﻿Phylogenetic analyses

The quality of the amplified nucleotide sequences was checked and combined using SeqMan v.7.1.0 and reference sequences were retrieved from the National Center for Biotechnology Information (NCBI), based on recent publications on the genus *Diaporthe* (Guarnaccia et al. 2018; Yang et al. 2018, 2020, 2021; Cao et al. 2022). Sequences were aligned using MAFFT v.6 (Katoh and Toh 2010) and corrected manually using Bioedit 7.0.9.0 (Hall 1999). The best-fit nucleotide substitution models for each gene were selected using jModelTest v.2.1.7 (Darriba et al. 2012) under the Akaike Information Criterion.

The phylogenetic analyses of the combined gene regions were performed using Maximum Likelihood (ML) and Bayesian Inference (BI) methods. ML was conducted using PhyML v.3.0 (Guindon et al. 2010), with 1000 bootstrap replicates, while BI was performed using a Markov Chain Monte Carlo (MCMC) algorithm in MrBayes v.3.0 (Ronquist and Huelsenbeck 2003). Two MCMC chains, started from random trees for 1,000,000 generations and trees, were sampled every 100^th^ generation, resulting in a total of 10,000 trees. The first 25% of trees were discarded as burn-in of each analysis. Branches with significant Bayesian Posterior Probabilities (BPP) were estimated in the remaining 7500 trees. Phylogenetic trees were viewed with FigTree v.1.3.1 (Rambaut and Drummond 2010) and processed by Adobe Illustrator CS5. The nucleotide sequence data of the new taxa were deposited in GenBank (Table 1). The multilocus sequence alignments were deposited in TreeBASE (www.treebase.org) as accession S30780.

**Table 1.** Isolates and GenBank accession numbers used in the phylogenetic analyses of *Diaporthe*.

Species	Isolate	GenBank accession numbers				
		ITS	*cal*	*his3*	*tef1*	*tub2*
*Diaportheacaciigena*	CBS 129521	KC343005	KC343247	KC343489	KC343731	KC343973
*Diaportheacericola*	MFLUCC 17-0956	KY964224	KY964137	NA	KY964180	KY964074
*Diaportheacerigena*	CFCC 52554	MH121489	MH121413	MH121449	MH121531	NA
*Diaportheacuta*	PSCG 047	MK626957	MK691125	MK726161	MK654802	MK691225
*Diaportheacutispora*	LC6161	KX986764	KX999274	KX999235	KX999155	KX999195
*Diaportheaestuarium*	BRIP 59930a	OM918686	NA	NA	OM960595	OM960613
*Diaporthealangii*	CFCC 52556	MH121491	MH121415	MH121451	MH121533	MH121573
*Diaporthealbosinensis*	CFCC 53066	MK432659	MK442979	MK443004	MK578133	MK578059
*Diaporthealleghaniensis*	CBS 495.72	KC343007	KC343249	KC343491	KC343733	KC343975
*Diaportheambigua*	CBS 114015	KC343010	KC343252	KC343494	KC343736	KC343978
*Diaportheampelina*	STE-U 2660	AF230751	AY745026	NA	AY745056	JX275452
*Diaportheamygdali*	CBS 126679	MH864208	KC343264	KC343506	KC343748	KC343990
*Diaportheamygdali* syn. *D.chongqingensis*	PSCG 435	MK626916	MK691209	MK726257	MK654866	MK691321
*Diaportheamygdali* syn. *D.fusicola*	CGMCC 3.17087	KF576281	KF576233	NA	KF576256	KF576305
*Diaportheamygdali* syn. *D.garethjonesii*	MFLUCC 12-0542a	KT459423	KT459470	NA	KT459457	KT459441
*Diaportheamygdali* syn. *D.kadsurae*	CFCC 52586	MH121521	MH121439	MH121479	MH121563	MH121600
*Diaportheamygdali* syn. *D.mediterranea*	SAUCC194.111	MT822639	MT855718	MT855606	MT855836	MT855951
*Diaportheamygdali* syn. *D.ovoicicola*	CGMCC 3.17093	KF576265	KF576223	NA	KF576240	KF576289
*Diaportheamygdali* syn. *D.sterilis*	CBS 136969	KJ160579	KJ160548	MF418350	KJ160611	KJ160528
*Diaportheamygdali* syn. *D.ternstroemiae*	CGMCC 3.15183	KC153098	NA	NA	KC153089	NA
*Diaportheanacardii*	CBS 720.97	KC343024	KC343266	KC343508	KC343750	KC343992
*Diaportheangelicae*	CBS 111592	KC343027	KC343269	KC343511	KC343753	KC343995
*Diaportheannellsiae*	BRIP 59731a	OM918687	NA	NA	OM960596	OM960614
*Diaportheapiculata*	CFCC 53068	MK432651	MK442973	MK442998	MK578127	MK578054
*Diaportheaquatica*	IFRDCC 3051	JQ797437	NA	NA	NA	NA
*Diaporthearctii*	DP0482	KJ590736	KJ612133	KJ659218	KJ590776	KJ610891
*Diaporthearecae*	CBS 161.64	KC343032	KC343274	KC343516	KC343758	KC344000
*Diaporthearengae*	CBS 114979	KC343034	KC343276	KC343518	KC343760	KC344002
*Diaportheaseana*	MFLUCC 12-0299a	KT459414	KT459464	NA	KT459448	KT459432
*Diaportheasheicola*	CBS 136967	KJ160562	KJ160542	NA	KJ160594	KJ160518
*Diaportheaspalathi*	CBS 117169	KC343036	KC343278	KC343520	KC343762	KC344004
*Diaportheaustralafricana*	CBS 111886	KC343038	KC343280	KC343522	KC343764	KC344006
*Diaportheaustraliana*	CBS 146457	MN708222	NA	NA	MN696522	MN696530
*Diaportheaustralpacifica*	BRIP 60163d	OM918688	NA	NA	OM960597	OM960615
*Diaporthebaccae*	CBS 136972	KJ160565	MG281695	MF418264	KJ160597	MF418509
*Diaporthebatatas*	CBS 122.21	KC343040	KC343282	KC343524	KC343766	KC344008
*Diaporthebauhiniae*	CFCC 53071	MK432648	MK442970	MK442995	MK578124	MK578051
*Diaporthebeasleyi*	BRIP 59326a	OM918689	NA	NA	OM960598	OM960616
*Diaporthebeilharziae*	BRIP 54792	JX862529	NA	NA	JX862535	KF170921
*Diaporthebenedicti*	SBen914	KM669929	KM669862	NA	KM669785	NA
*Diaporthebetulae*	CFCC 50469	KT732950	KT732997	KT732999	KT733016	KT733020
*Diaporthebetulicola*	CFCC 51128	KX024653	KX024659	KX024661	KX024655	KX024657
*Diaporthebetulina*	CFCC 52560	MH121495	MH121419	MH121455	MH121537	MH121577
*Diaporthebiconispora*	ZJUD62	KJ490597	NA	KJ490539	KJ490476	KJ490418
*Diaporthebiguttulata*	ZJUD47	KJ490582	NA	KJ490524	KJ490461	KJ490403
	CFCC 52584	MH121519	MH121437	MH121477	MH121561	MH121598
*Diaporthebohemiae*	CBS 143347	MG281015	MG281710	MG281361	MG281536	MG281188
*Diaporthebounty*	BRIP 59361a	OM918690	NA	NA	OM960599	OM960617
*Diaporthebrasiliensis*	CBS 133183	KC343042	KC343284	KC343526	KC343768	KC344010
*Diaporthebreyniae*	CBS 148910	ON400846	ON409189	ON409187	ON409188	ON409186
*Diaporthebrumptoniae*	BRIP 59403a	OM918702	NA	NA	OM960611	OM960629
*Diaporthecaatingaensis*	URM7486	KY085927	KY115597	KY115605	KY115603	KY115600
*Diaporthecamelliae-sinensis*	SAUCC194.92	MT822620	MT855699	MT855588	MT855932	MT855817
*Diaporthecamelliae-oleiferae*	HNZZ027	MZ509555	MZ504685	MZ504696	MZ504707	MZ504718
*Diaporthecanthii*	CPC 19740	JX069864	KC843174	NA	KC843120	KC843230
*Diaporthecarriae*	BRIP 59932a	OM918691	NA	NA	OM960600	OM960618
*Diaporthecaryae*	CFCC 52563	MH121498	MH121422	MH121458	MH121540	MH121580
*Diaporthecassines*	CPC 21916	KF777155	NA	NA	KF777244	NA
*Diaporthecaulivora*	CBS 127268	MH864501	KC343287	KC343529	KC343771	KC344013
*Diaporthecelticola*	CFCC 53074	MK573948	MK574587	MK574603	MK574623	MK574643
*Diaporthecercidis*	CFCC 52565	MH121500	MH121424	MH121460	MH121542	MH121582
*Diaporthechamaeropis*	CBS 454.81	KC343048	KC343290	KC343532	KC343774	KC344016
*Diaporthecharlesworthii*	BRIP 54884m	KJ197288	NA	NA	KJ197250	KJ197268
*Diaporthechiangmaiensis*	MFLU 18-1305	OK393702	NA	NA	OL439482	OK490918
*Diaporthechrysalidocarpi*	SAUCC194.35	MT822563	MT855646	MT855532	MT855760	MT855876
*Diaporthecichorii*	MFLUCC 17-1023	KY964220	KY964133	NA	KY964176	KY964104
*Diaporthecinnamomi*	CFCC 52569	MH121504	NA	MH121464	MH121546	MH121586
*Diaporthecissampeli*	CPC 27302	KX228273	NA	KX228366	NA	KX228384
*Diaporthecitri*	AR3405	KC843311	KC843157	KJ420881	KC843071	KC843187
*Diaporthechensiensis*	CFCC 52567	MH121502	MH121426	MH121462	MH121544	MH121584
*Diaporthecitriasiana*	CGMCC 3.15224	JQ954645	KC357491	KJ490515	JQ954663	KC357459
*Diaporthecitrichinensis*	CGMCC 3.15225	JQ954648	KC357494	KJ420880	JQ954666	KJ490396
*Diaporthecollariana*	MFLU 17-2770	MG806115	MG783042	NA	MG783040	MG783041
*Diaporthecompactum*	LC3083	KP267854	NA	KP293508	KP267928	KP293434
*Diaportheconica*	CFCC 52571	MH121506	MH121428	MH121466	MH121548	MH121588
*Diaportheconvolvuli*	CBS 124654	KC343054	KC343296	KC343538	KC343780	KC344022
*Diaporthecoryli*	CFCC 53083	MK432661	MK442981	MK443006	MK578135	MK578061
*Diaporthecrotalariae*	CBS 162.33	MH855395	JX197439	KC343540	GQ250307	KC344024
*Diaporthecrousii*	CAA 823	MK792311	MK883835	MK871450	MK828081	MK837932
*Diaporthecucurbitae*	DAOM 42078	KM453210	NA	KM453212	KM453211	KP118848
*Diaporthecuppatea*	CBS 117499	MH863021	KC343299	KC343541	KC343783	KC344025
*Diaporthecynaroidis*	CBS 122676	KC343058	KC343300	KC343542	KC343784	KC344026
*Diaporthecytosporella*	FAU461	KC843307	KC843141	MF418283	KC843116	KC843221
*Diaporthediospyricola*	CPC 21169	KF777156	NA	NA	NA	NA
*Diaporthediscoidispora*	ZJUD89	KJ490624	NA	KJ490566	KJ490503	KJ490445
*Diaporthedorycnii*	MFLUCC 17-1015	KY964215	NA	NA	KY964171	KY964099
*Diaporthedrenthii*	CBS 146453	MN708229	NA	NA	MN696526	MN696537
*Diaporthedurionigena*	VTCC 930005	MN453530	NA	NA	MT276157	MT276159
*Diaportheelaeagni-glabrae*	LC4802	KX986779	KX999281	KX999251	KX999171	KX999212
*Diaportheendophytica*	CBS 133811	KC343065	KC343307	KC343549	KC343791	KC344033
*Diaportheeres*	AR5193	KJ210529	KJ434999	KJ420850	KJ210550	KJ420799
*Diaportheetinsideae*	BRIP 64096a	OM918692	NA	NA	OM960601	OM960619
*Diaportheeucalyptorum*	CBS 132525	MH305525	NA	NA	NA	NA
*Diaporthefoeniculacea*	CBS 111553	KC343101	KC343343	KC343585	KC343827	KC344069
*Diaporthefraxini-angustifoliae*	BRIP 54781	JX862528	NA	NA	JX862534	KF170920
*Diaporthefraxinicola*	CFCC 52582	MH121517	MH121435	NA	MH121559	NA
*Diaporthefructicola*	MAFF 246408	LC342734	LC342738	LC342737	LC342735	LC342736
*Diaporthefulvicolor*	PSCG 051	MK626859	MK691132	MK726163	MK654806	MK691236
*Diaportheganjae*	CBS 180.91	KC343112	KC343354	KC343596	KC343838	KC344080
*Diaportheganzhouensis*	CFCC 53087	MK432665	MK442985	MK443010	MK578139	MK578065
*Diaporthegoulteri*	BRIP 55657a	KJ197290	NA	NA	KJ197252	KJ197270
*Diaporthegossiae*	BRIP 59730a	OM918693	NA	NA	OM960602	OM960620
*Diaporthegrandiflori*	SAUCC194.84	MT822612	MT855691	MT855580	MT855809	MT855924
*Diaporthegriceae*	BRIP 67014a	OM918694	NA	NA	OM960603	OM960621
*Diaportheguangxiensis*	JZB320087	MK335765	MK736720	NA	MK500161	MK523560
*Diaporthegulyae*	BRIP 54025	JF431299	NA	NA	JN645803	KJ197271
*Diaportheguttulata*	CGMCC 3.20100	MT385950	MW022470	MW022491	MT424685	MT424705
***Diaporthehainanenesis***	**HNCM049**	**OR647684**	**NA**	**OR671936**	**OR671944**	**OR671952**
	**HNCM050**	**OR647685**	**NA**	**OR671937**	**OR671945**	**OR671953**
	**HNCM051**	**OR647686**	**NA**	**OR671938**	**OR671946**	**OR671954**
	**HNCM052**	**OR647687**	**NA**	**OR671939**	**OR671947**	**OR671955**
*Diaporthehelianthi*	CBS 592.81	KC343115	KC343357	KC343599	KC343841	KC344083
*Diaportheheliconiae*	SAUCC194.77	MT822605	MT855684	MT855573	MT855802	MT855917
*Diaportheheterophyllae*	CPC 26215	MG600222	MG600218	MG600220	MG600224	MG600226
*Diaportheheterostemmatis*	SAUCC194.85	MT822613	MT855692	MT855581	MT855810	MT855925
*Diaporthehickoriae*	CBS 145.26	KC343118	KC343360	KC343620	KC343844	KC344086
*Diaporthehispaniae*	CBS 143351	MG281123	MG281820	MG281471	MG281644	MG281296
*Diaporthehongkongensis*	CBS 115448	KC343119	KC343361	KC343603	KC343845	KC344087
*Diaporthehowardiae*	BRIP 59697a	OM918695	NA	NA	OM960604	OM960622
*Diaporthehubeiensis*	JZB320123	MK335809	MK500235	NA	MK523570	MK500148
*Diaporthehunanensis*	HNZZ023	MZ509550	MZ504680	MZ504691	MZ504702	MZ504713
*Diaportheincompleta*	LC6754	KX986794	KX999289	KX999265	KX999186	KX999226
*Diaportheinconspicua*	CBS 133813	KC343123	KC343365	KC343607	KC343849	KC344091
*Diaportheinfecunda*	CBS 133812	KC343126	KC343368	KC343610	KC343852	KC344094
*Diaportheirregularis*	CGMCC 3.20092	MT385951	MT424721	NA	MT424686	MT424706
*Diaportheisoberliniae*	CPC 22549	KJ869190	NA	NA	NA	KJ869245
*Diaporthejuglandicola*	CFCC 51134	KU985101	KX024616	KX024622	KX024628	KX024634
*Diaporthekochmanii*	BRIP 54033	JF431295	NA	NA	JN645809	NA
*Diaporthekongii*	BRIP 54031	JF431301	NA	NA	JN645797	KJ197272
*Diaporthekrabiensis*	MFLUCC 17-2481	MN047100	NA	NA	MN433215	MN431495
*Diaporthelenispora*	CGMCC 3.20101	MT385952	MW022472	MW022493	MT424687	MT424707
*Diaporthelitchicola*	BRIP 54900	JX862533	NA	NA	JX862539	KF170925
*Diaporthelitchii*	SAUCC194.22	MT822550	MT855635	MT855519	MT855747	MT855863
*Diaporthelithocarpi*	CGMCC 3.15175	KC135104	KF576235	NA	KC153095	KF576311
*Diaporthelongicolla*	FAU599	KJ590728	KJ612124	KJ659188	KJ590767	KJ610883
*Diaporthelongispora*	CBS 194.36	MH855769	KC343377	KC343619	KC343861	KC344103
*Diaporthelovelaceae*	BRIP 60163a	OM918696	NA	NA	OM960605	OM960623
*Diaporthelusitanicae*	CBS 123212	MH863279	KC343378	KC343620	KC343862	KC344104
*Diaporthelutescens*	SAUCC194.36	MT822564	MT855647	MT855533	MT855761	MT855877
*Diaporthemacadamiae*	CBS 146455	MN708230	NA	NA	MN696528	MN696539
*Diaporthemacintoshii*	BRIP 55064a	KJ197289	NA	NA	KJ197251	KJ197269
*Diaporthemalorum*	CAA 734	KY435638	KY435658	KY435648	KY435627	KY435668
*Diaporthemarina*	MFLU 17-2622	MN047102	NA	NA	NA	NA
*Diaporthemasirevicii*	BRIP 54256	KJ197276	NA	NA	KJ197238	KJ197256
*Diaporthemayteni*	CBS 133185	KC343139	KC343381	KC343623	KC343865	KC344107
*Diaporthemaytenicola*	CPC 21896	KF777157	NA	NA	NA	KF777250
*Diaporthemclennaniae*	BRIP 60072a	OM918697	NA	NA	OM960606	OM960624
*Diaporthemelastomatis*	SAUCC194.55	MT822583	MT855664	MT855551	MT855780	MT855896
*Diaporthemelonis*	CBS 435.87	KC343141	KC343383	KC343625	KC343867	KC344109
*Diaporthemiddletonii*	BRIP 54884e	KJ197286	NA	NA	KJ197248	KJ197266
*Diaportheminima*	CGMCC 3.20097	MT385953	MT424722	MW022496	MT424688	MT424708
*Diaportheminusculata*	CGMCC 3.20098	MT385957	MW022475	MW022499	MT424692	MT424712
*Diaporthemiriciae*	BRIP 54736j	KJ197282	NA	NA	KJ197244	KJ197262
*Diaporthemonetii*	MF-Ha18-048	MW008493	MZ671938	MZ671964	MW008515	MW008504
*Diaporthemoriniae*	BRIP 60190a	OM918698	NA	NA	OM960607	OM960625
*Diaporthemultigutullata*	CFCC 53095	MK432645	MK442967	MK442992	MK578121	MK578048
*Diaporthemusigena*	CBS 129519	KC343143	KC343385	KC343267	KC343869	KC344111
*Diaporthemyracrodruonis*	URM 7972	MK205289	MK205290	NA	MK213408	MK205291
*Diaportheneoarctii*	CBS 109490	KC343145	KC343387	KC343629	KC343871	KC344113
*Diaportheneoraonikayaporum*	MFLUCC 14-1136	KU712449	KU749356	NA	KU749369	KU743988
*Diaporthenorfolkensis*	BRIP 59718a	OM918699	NA	NA	OM960608	OM960626
*Diaporthenothofagi*	BRIP 54801	JX862530	NA	NA	JX862536	KF170922
*Diaporthenovem*	CBS 127269	KC343155	KC343397	KC343639	KC343881	KC344123
*Diaportheocoteae*	CPC 26217	KX228293	NA	NA	NA	KX228388
*Diaportheoraccinii*	LC3166	KP267863	NA	KP293517	KP267937	KP293443
*Diaportheovalispora*	ZJUD93	KJ490628	NA	KJ490570	KJ490507	KJ490449
*Diaportheoxe*	CBS 133186	KC343164	KC343406	KC343648	KC343890	KC344132
*Diaporthepadina*	CFCC 52590	MH121525	MH121443	MH121483	MH121567	MH121604
*Diaporthepandanicola*	MFLUCC 17-0607	MG646974	NA	NA	NA	MG646930
*Diaportheparanensis*	CBS 133184	KC343171	KC343413	KC343655	KC343897	KC344139
*Diaportheparapterocarpi*	CPC 22729	KJ869138	NA	NA	NA	KJ869248
*Diaportheparvae*	PSCG 035	MK626920	MK691169	MK726211	MK654859	MK691249
*Diaporthepascoei*	BRIP 54847	JX862538	NA	NA	JX862538	KF170924
*Diaporthepassiflorae*	CPC 19183	JX069860	KY435644	KY435654	KY435623	KY435674
*Diaporthepassifloricola*	CPC 27480	KX228292	NA	KX228367	NA	KX228387
*Diaporthepenetriteum*	LC3215	KP267879	NA	KP293532	KP267953	NA
*Diaportheperjuncta*	CBS 109745	KC343172	KC343414	KC343656	KC343898	KC344140
*Diaportheperseae*	CBS 151.73	KC343173	KC343415	KC343657	KC343899	KC343141
*Diaporthepescicola*	MFLUCC 16-0105	KU557555	KU557603	NA	KU557623	KU557579
*Diaporthephaseolorum*	AR4203	KJ590738	KJ612135	KJ659220	KJ590739	KJ610893
*Diaportheplatzii*	BRIP 60353a	OM918700	NA	NA	OM960609	OM960627
*Diaporthephillipsii*	CAA 817	MK792305	MK883831	MK871445	MK828076	MN000351
*Diaporthepodocarpi-macrophylli*	LC6155	KX986774	KX999278	KX999246	KX999167	KX999207
*Diaporthepometiae*	SAUCC194.72	MT822600	MT855679	MT855568	MT855797	MT855912
*Diaporthepseudoalnea*	CFCC 54190	MZ727037	MZ753468	MZ781302	MZ816343	MZ753487
***Diaporthepseudofoliicola***	**HNCM045**	**OR647680**	**NA**	**OR671932**	**OR671940**	**OR671948**
	**HNCM046**	**OR647681**	**NA**	**OR671933**	**OR671941**	**OR671949**
	**HNCM047**	**OR647682**	**NA**	**OR671934**	**OR671942**	**OR671950**
	**HNCM048**	**OR647683**	**NA**	**OR671935**	**OR671943**	**OR671951**
*Diaporthepseudomangiferae*	CBS 101339	KC343181	KC343423	KC343665	KC343907	KC344149
*Diaporthepseudophoenicicola*	CBS 176.77	KC343183	KC343425	KC343667	KC343909	KC344151
*Diaporthepsoraleae*	CPC 21634	KF777158	NA	NA	KF777245	KF777251
*Diaporthepsoraleae-pinnatae*	CPC 21638	KF777159	NA	NA	NA	KF777252
*Diaporthepterocarpi*	CPC 22729	JQ619899	JX197451	NA	JX275416	JX275460
*Diaporthepterocarpicola*	MFLUCC 10-0580a	JQ619887	JX197433	NA	JX275403	JX275441
*Diaporthepungensis*	SAUCC194.112	MT822640	MT855719	MT855607	MT855837	MT855952
*Diaporthepyracanthae*	CAA483	KY435635	KY435656	KY435645	KY435625	KY435666
*Diaportheracemosae*	CPC 26646	MG600223	MG600219	MG600221	MG600225	MG600227
*Diaportheraonikayaporum*	CBS 133182	KC343188	KC343430	KC343672	KC343914	KC344156
*Diaportheravennica*	MFLUCC 16-0997	NA	NA	NA	MT394670	NA
*Diaportherhodomyrti*	CFCC 53101	MK432643	MK442965	MK442990	MK578119	MK578046
*Diaportherhusicola*	CPC 18191	JF951146	KC843124	NA	KC843100	KC843205
*Diaportherosae*	MFLUCC 17-2658	MG828894	MG829273	NA	NA	MG843878
*Diaportherosiphthora*	COAD 2914	MT311197	MT313691	NA	MT313693	NA
*Diaportherossmaniae*	CAA 762	MK792290	MK883822	MK871432	MK828063	MK837914
*Diaportherostrata*	CFCC 50062	KP208847	KP208849	KP208851	KP208853	KP208855
*Diaportherudis*	AR3422	KC843331	KC843146	NA	KC843090	KC843177
*Diaporthesaccarata*	CBS 116311	KC343190	KC343432	KC343674	KC343916	KC344158
*Diaporthesackstonii*	BRIP 54669b	KJ197287	NA	NA	KJ197249	KJ197267
*Diaporthesalicicola*	BRIP 54825	JX862531	NA	NA	JX862537	KF170923
*Diaporthesambucusii*	CFCC 51986	KY852495	KY852499	KY852503	KY852507	KY852511
*Diaportheschimae*	CFCC 53103	MK442640	MK442962	MK442987	MK578116	MK578043
*Diaportheschini*	CBS 133181	KC343191	KC343433	KC343675	KC343917	KC344159
*Diaportheschisandrae*	CFCC 51988	KY852497	KY852501	KY852505	KY852509	KY852513
*Diaportheschoeni*	MFLU 15-1279	KY964226	KY964139	NA	KY964182	KY964109
*Diaporthesclerotioides*	CBS 296.67	MH858974	KC343435	KC343677	KC343919	KC344161
*Diaporthesearlei*	BRIP 66528	MN708231	NA	NA	NA	MN696540
*Diaporthesennae*	CFCC 51636	KY203724	KY228875	NA	KY228885	KY228891
*Diaporthesennicola*	CFCC 51634	KY203722	KY228873	KY228879	KY228883	KY228889
*Diaportheserafiniae*	BRIP 55665a	KJ197274	NA	NA	KJ197236	KJ197254
*Diaportheshaanxiensis*	CFCC 53106	MK432654	MK442976	MK443001	MK578130	NA
*Diaportheshawiae*	BRIP 64534a	OM918701	NA	NA	OM960610	OM960628
*Diaporthesiamensis*	MFLUCC 10-0573a	JQ619879	JX197423	NA	JX275393	JX275429
*Diaporthesilvicola*	CFCC 54191	MZ727041	MZ753472	MZ753481	MZ816347	MZ753491
*Diaporthesojae*	FAU635	KJ590719	KJ612116	KJ659208	KJ590762	KJ610875
*Diaporthespinosa*	PSCG 383	MK626849	MK691129	MK726156	MK654811	MK691234
*Diaporthestictica*	CBS 370.54	KC343212	KC343454	KC343696	KC343938	KC344180
*Diaporthesubclavata*	ZJUD95	KJ490630	NA	KJ490572	KJ490509	KJ490451
*Diaporthesubcylindrospora*	KUMCC 17-0151	MG746629	NA	NA	MG746630	MG746631
*Diaporthesubellipicola*	KUMCC 17-0153	MG746632	NA	NA	MG746633	MG746634
*Diaporthesubordinaria*	CBS 464.90	KC343214	KC343456	KC343698	KC343940	KC344182
*Diaporthetaoicola*	MFLUCC 16-0117	KU557567	NA	NA	KU557635	KU557591
*Diaporthetectonae*	MFLUCC 12-0777	KU712430	KU749345	NA	KU749359	KU743977
*Diaporthetectonendophytica*	MFLUCC 13-0471	KU712439	KU749354	NA	KU749367	KU743986
*Diaporthetectonigena*	MFLUCC 12-0767	KX986782	KX999284	KX999254	KX999174	KX999214
*Diaportheterebinthifolii*	CBS 133180	KC343216	KC343458	KC343700	KC343942	KC344184
*Diaporthethunbergii*	MFLUCC 10-0576a	JQ619893	JX197440	NA	JX275409	JX275449
*Diaporthethunbergiicola*	MFLUCC 12-0033	KP715097	NA	NA	KP715098	NA
*Diaporthetibetensis*	CFCC 51999	MF279843	MF279888	MF279828	MF279858	MF279873
*Diaporthetulliensis*	BRIP 62248a	KR936130	NA	NA	KR936133	KR936132
*Diaporthetrevorrowii*	BRIP 70737a	OM918703	NA	NA	OM960612	OM960630
*Diaportheueckerae*	FAU656	KJ590726	KJ612122	KJ659215	KJ590747	KJ610881
*Diaportheukurunduensis*	CFCC 52592	MH121527	MH121445	MH121485	MH121569	NA
*Diaportheundulata*	LC6624	KX986798	NA	KX999269	KX999190	KX999230
*Diaportheunshiuensis*	CFCC 52594	MH121529	MH121447	MH121487	MH121571	MH121606
	CFCC 52595	MH121530	MH121448	MH121488	MH121572	MH121607
*Diaporthevaccinii*	CBS 160.32	KC343228	KC343470	KC343712	KC343954	KC343196
*Diaporthevangoghii*	MF-Ha18-045	MW008491	MZ671936	MZ671962	MW008513	MW008502
*Diaporthevangueriae*	CBS 137985	KJ869137	NA	NA	NA	KJ869247
*Diaporthevawdreyi*	BRIP 57887a	KR936126	NA	NA	KR936129	KR936128
*Diaporthevelutina*	LC4421	KX986790	NA	KX999261	KX999182	KX999223
*Diaportheverniciicola*	CFCC 53109	MK573944	MK574583	MK574599	MK574619	MK574639
*Diaportheviniferae*	JZB320071	MK341551	MK500119	NA	MK500107	MK500112
*Diaporthevirgiliae*	CMW 40748	KP247556	NA	NA	NA	KP247575
*Diaporthexishuangbanica*	LC6707	KX986783	NA	KX999255	KX999175	KX999216
*Diaporthexunwuensis*	CFCC 53085	MK432663	MK442983	MK443008	MK578137	MK578063
*Diaportheyunnanensis*	LC6168	KX986796	KX999290	KX999267	KX999188	KX999228
*Diaporthezaobaisu*	PSCG 031	MK626922	NA	MK726207	MK654855	MK691245
*Diaporthellacorylina*	CBS 121124	KC343004	KC343246	KC343488	KC343730	KC343972

Note: NA, not applicable. Strains in this study are marked in bold.

## ﻿Results

### ﻿Phylogenetic analyses

**Figure 1.**
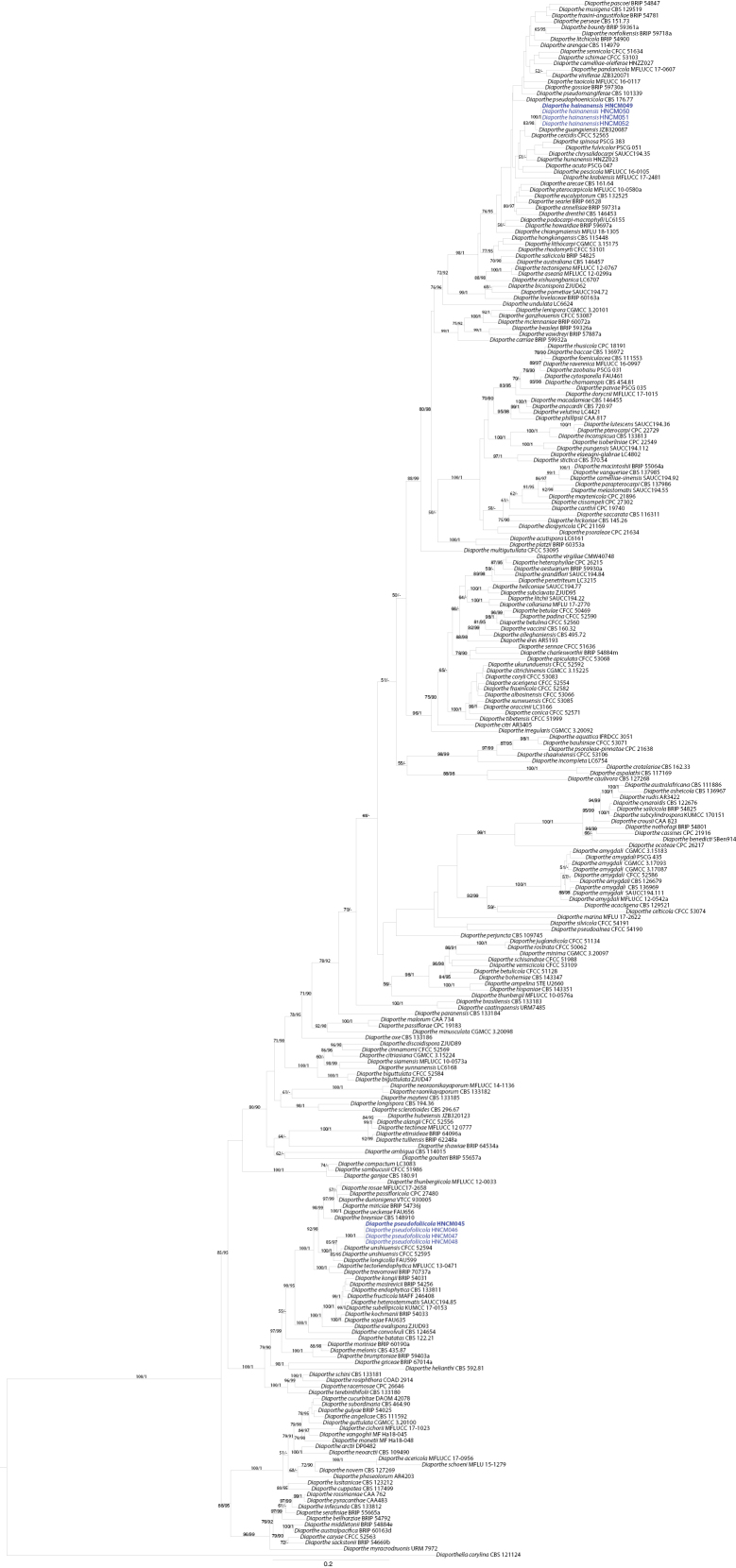
Phylogram of *Diaporthe* resulting from a Maximum Likelihood analysis, based on combined ITS, *cal*, *his3*, *tef1* and *tub2*. Numbers above the branches indicate ML bootstraps (left, ML BS ≥ 50%) and Bayesian Posterior Probabilities (right, BPP ≥ 0.9). The tree is rooted with *Diaporthellacorylina*. Isolates in the current study are in blue. “-” indicates ML BS < 50% or BI PP < 0.9.

The five-gene sequence dataset (ITS, *cal*, *his3*, *tef1* and *tub2*) was analysed to infer the interspecific relationships within *Diaporthe*. The dataset consisted of 259 sequences including the outgroup taxon, *Diaporthellacorylina* (CBS 121124). A total of 2909 characters including gaps (528 for ITS, 608 for *cal*, 563 for *his3*, 646 for *tef1* and 564 for *tub2*) were included in the phylogenetic analysis. The best nucleotide substitution model for ITS, *his3* and *tub2* was TrN+I+G, while HKY+I+G was selected for both *cal* and *tef1*. The topologies resulting from ML and BI analyses of the concatenated dataset were congruent (Fig. 1). According to the phylogenetic tree, *D.hainanensis* and *D.pseudofoliicola* are new to science, based on the distinct and well-supported molecular phylogenetic placement with their closest described relatives. Phylogenetically, *D.pseudofoliicola* clustered together with *D.longicolla* and *D.unshiuensis*. *Diaporthehainanensis* clustered together with *D.cercidis* and *D.guangxiensis*.

### ﻿Taxonomy

#### ﻿ Diaporthe hainanensis

Taxon classificationFungiDiaporthalesDiaporthaceae

Q. Yang sp. nov.

83930BD8-3D80-589A-999F-7AE9D5CBC803

MycoBank No: 848328

Fig. 2

##### Diagnosis.

Distinguished from *D.cercidis* in narrower alpha conidia; from *D.guangxiensis* in shorter beta conidia.

##### Etymology.

In reference to the Hainan Province, from where the fungus was first collected.

##### Description.

***Asexual morph***: Conidiomata on PNA pycnidial, globose or rostrated, black, erumpent in tissue, erumpent at maturity, 450–600 μm diam., often with pale yellowish conidial drops exuding from the ostioles. ***Conidiophores*** reduced to conidiogenous cells. ***Conidiogenous cells*** (10.5–)14.5–20(–21.5) × 1.4–1.8 μm (n = 30), aseptate, cylindrical, phialidic, straight or slightly curved. ***Alpha conidia*** (5.5–)7–8(–8.5) × 2.1–2.9 μm (n = 30), aseptate, hyaline, ellipsoidal, biguttulate. ***Beta conidia*** (21.5–)23–25 × 1.1 µm (n = 30), hyaline, aseptate, filiform, sinuous at one end, eguttulate.

**Figure 2.**
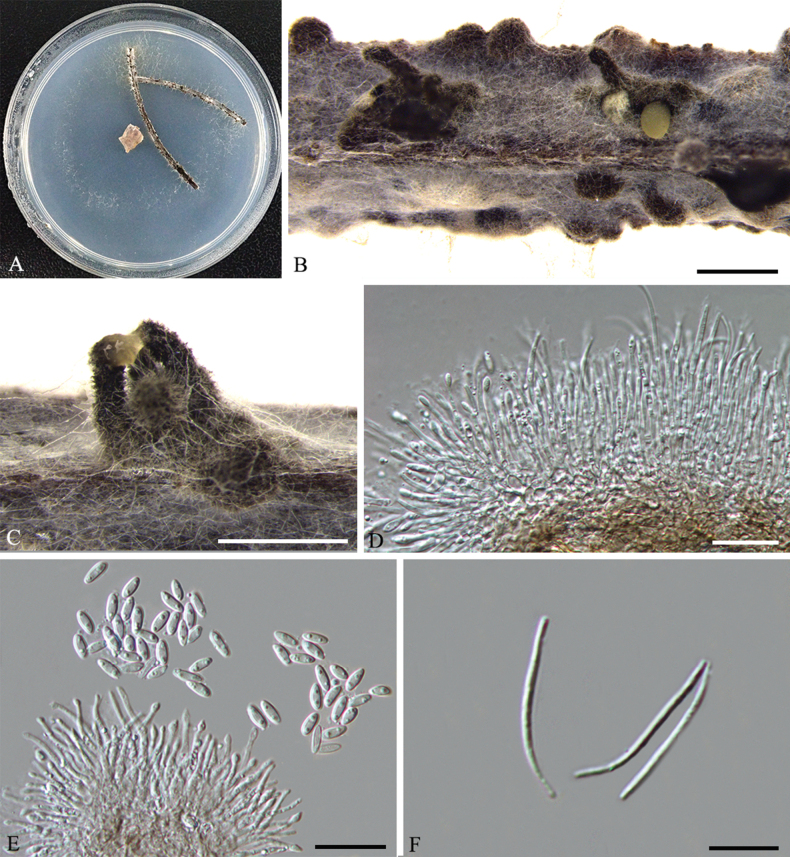
*Diaporthehainanensis* (HNCM049) **A** culture on PNA**B, C** conidiomata **D** conidiogenous cells **E** alpha conidia **F** beta conidia. Scale bars: 500 μm (**B, C**); 10 μm (**C, F**), 20 μm (**E**).

##### Culture characters.

Culture incubated on PNA at 25 °C, originally white, fluffy aerial mycelium, becoming pale yellow with age, with visible solitary conidiomata pine needles after 15 days.

##### Specimens examined.

China. Hainan Province: Chengmai County, on leaves of *Camelliaoleifera*, 19°34'10"N, 110°18'09"E, 25 July 2022, *Q. Yang* (holotype CSUFT055; ex-type living culture: HNCM049; other living cultures: HNCM050, HNCM051 and HNCM052).

##### Notes.

Four isolates representing *D.hainanensis* cluster in a well-supported clade (ML/BI = 100/1) and appear most closely related to *D.cercidis* on *Cercischinensis* and *D.guangxiensis* on *Macadamia* sp. *Diaporthehainanensis* can be distinguished from *D.cercidis*, based on ITS, *his3*, *tef1*and *tub2* loci (13/458 in ITS, 5/455 in *his3*, 33/341 in *tef1* and 5/401 in *tub2*); from *D.guangxiensis*, based on ITS, *tef1* and *tub2* loci (5/457 in ITS, 2/339 in *tef1* and 16/403 in *tub2*). Morphologically, *D.hainanensis* differs from *D.cercidis* in narrower alpha conidia (2.1–2.9 μm vs. 3–3.5 μm) (Yang et al. 2018); from *D.guangxiensis* in shorter beta conidia (23–25 μm vs. 20–32 μm) (Manawasinghe et al. 2019).

#### ﻿ Diaporthe pseudofoliicola

Taxon classificationFungiDiaporthalesDiaporthaceae

Q. Yang sp. nov.

054C182C-E989-566D-B604-3EF72EBC4C53

MycoBank No: 848327

Fig. 3

##### Diagnosis.

Distinguished from *D.longicolla* in having smaller alpha conidia; from *D.unshiuensis* in having narrower conidiophores.

##### Etymology.

The epithet “*pseudofoliicola*” refers to its habitat similar to *Diaporthefoliicola*.

##### Description.

***Asexual morph***: Conidiomata on PDA pycnidial, 190–330 μm in diam., superficial, scattered on PDA, dark brown to black, globose, solitary or clustered in groups of 1–3 pycnidia. Pale yellow conidial drops exuding from ostioles. ***Conidiophores*** reduced to conidiogenous cells. ***Conidiogenous cells*** (10.5–)12.5–18(–22) × 1.3–1.5 μm (n = 30), phialidic, aseptate, cylindrical, straight, densely aggregated, terminal, slightly tapered towards the apex. ***Alpha conidia*** 5–6.5(–7) × 2.3–3.0 μm (n = 30), aseptate, hyaline, ellipsoidal to fusiform, biguttulate, both ends obtuse. ***Beta conidia*** (27.5–)30–33(–35.5) × 1.2–1.4 µm (n = 30), hyaline, aseptate, filiform, sinuous at one end, eguttulate.

**Figure 3.**
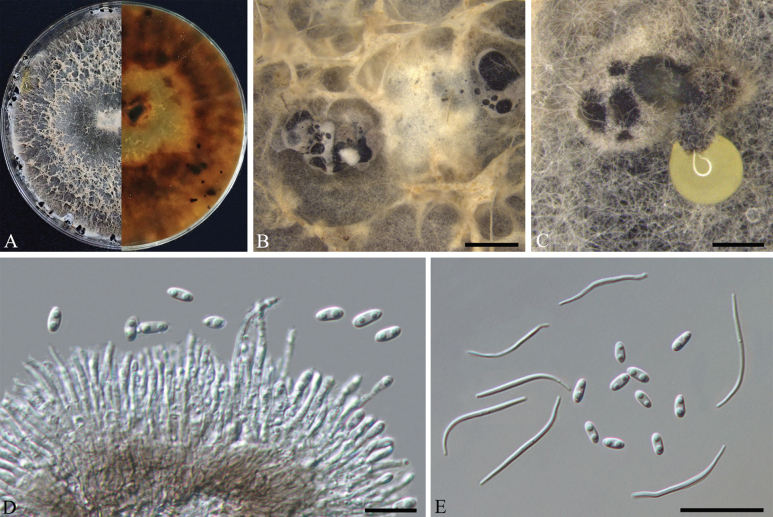
*Diaporthepseudofoliicola* (HNCM045) **A** culture on PDA**B, C** conidiomata **D** conidiogenous cells **E** alpha and beta conidia. Scale bars: 200 μm (**B, C**), 10 μm (**D**), 20 μm (**E**).

##### Culture characters.

Culture incubated on PDA at 25 °C, originally flat with white fluffy aerial mycelium, becoming pale brown due to pigment formation, with yellowish-cream conidial drops exuding from the ostioles after 20 days.

##### Specimens examined.

China. Hainan Province: Chengmai County, on leaves of *Camelliaoleifera*, 110°15'16"E, 19°23'20"N, 25 July 2022, *Q. Yang* (holotype CSUFT050; ex-type living culture: HNCM045; other living cultures: HNCM046, HNCM047 and HNCM048).

##### Notes.

Four isolates representing *D.pseudofoliicola* cluster in a well-supported clade (ML/BI = 100/1) and appear most closely related to *D.longicolla* on *Glycinemax* and *D.unshiuensis* on *Citrusunshiu*. *Diaporthepseudofoliicola* can be distinguished from *D.longicolla*, based on ITS, *tef1* and *tub2* loci (9/462 in ITS, 16/318 in *tef1* and 4/444 in *tub2*); from *D.unshiuensis*, based on *his3* and *tef1* loci (51/457 in *his3* and 17/318 in *tef1*). Morphologically, *D.pseudofoliicola* differs from *D.longicolla* in having smaller alpha conidia (5–6.5 × 2.3–3.0 μm vs. 6.9–7.2 × 1.6–2.8 μm) (Santos et al. 2011); from *D.unshiuensis* in having narrower conidiophores (1.3–1.5 μm vs. 1.4–2.6 μm) (Huang et al. 2015).

## ﻿Discussion

In this study, an important oil-tea tree species, *Camelliaoleifera* was investigated and *Camellia* leaf disease was found as a common disease in plantations in Hainan Province. Identification of our collections was conducted, based on isolates from symptomatic leaves of *C.oleifera* using five combined loci (ITS, *cal*, *his3*, *tef1* and *tub2*), as well as morphological characters. Two new *Diaporthe* species were described, i.e. *D.hainanensis* and *D.pseudofoliicola*.

According to the USDA Fungal-host interaction database, there are six records of *Diaporthe* species associated with *C.oleifera* (https://nt.ars-grin.gov/fungaldatabases; accessed on 18 Sep 2023). These records are related to the following six *Diaporthe* species: *D.eres*, *D.camelliae-oleiferae*, *D.hubeiensis*, *D.hunanensis*, *D.huangshanensis* and *D.sojae* (Zhou and Hou 2019; Yang et al. 2021). *Diaportheeres*, the type species of the genus, was described by Nitschke (1870) on *Ulmus* sp. collected in Germany, which has a widespread distribution and a broad host range as pathogens, endophytes or saprobes (Udayanga et al. 2014). *Diaportheeres* differs from *D.pseudofoliicola* and *D.hainanensis* in having wider alpha conidia (3–4 μm in *D.eres* vs. 2.3–3.0 μm in *D.pseudofoliicola* vs. 2.1–2.9 μm in *D.hainanensis*) (Gomes et al. 2013); *D.huangshanensis* differs from *D.pseudofoliicola* in having shorter beta conidia (19.5–30 μm vs. 30–33 μm); from *D.hainanensis* in having wider alpha conidia (2.7–4.5 μm vs. 2.1–2.9 μm) (Zhou and Hou 2019). Yang et al. (2021) recorded four *Diaporthe* species, *D.camelliae-oleiferae*, *D.hubeiensis*, *D.hunanensis* and *D.sojae*, which were isolated from *Camelliaoleifera* in Hunan Province and which can be distinguished from *D.pseudofoliicola* and *D.hainanensis*, based on DNA sequence data (Fig. 1).

As the species concept of *Diaporthe* has been greatly improved by using molecular data (Huang et al. 2015; Gao et al. 2016, 2017; Guarnaccia and Crous 2017; Guarnaccia et al. 2018; Yang et al. 2018, 2020, 2021; Manawasinghe et al. 2019; Guo et al. 2020; Jiang et al. 2021; Cao et al. 2022; Bai et al. 2023; Zhu et al. 2023), many new species have been discovered and reported in recent years. In this study, the *Diaporthe* isolates from *C.oleifera* were identified, based on sequence analysis and morphological characteristics. Future studies should focus on pathogenicity, epidemiology and fungicide sensitivity of the important plant fungal pathogen to develop effective management of *C.oleifera* disease and on the pathogenic molecular mechanism.

## Supplementary Material

XML Treatment for Diaporthe hainanensis

XML Treatment for Diaporthe pseudofoliicola

## ﻿Additional information

### Data availability

All of the data that support the findings of this study are available in the main text.
